# Phytosterol Depletion in Soybean Oil Using a Synthetic Silica Adsorbent

**DOI:** 10.3390/foods13193172

**Published:** 2024-10-06

**Authors:** Birgit Steiner-Zitzenbacher, Joaquín Velasco, Crispulo Gallegos, Maria-Victoria Ruiz-Méndez

**Affiliations:** 1 R&D Graz, Business Unit Nutrition, Fresenius Kabi Austria GmbH, 8055 Graz, Austria; birgit.steiner-zitzenbacher@fresenius-kabi.com; 2 Department of Characterization and Quality of Lipids, Instituto de la Grasa-CSIC, 41013 Seville, Spain; jvelasco@ig.csic.es; 3 Business Unit Nutrition, Fresenius Kabi Deutschland GmbH, 61352 Bad Homburg, Germany; crispulo.gallegos-montes@fresenius-kabi.com

**Keywords:** soybean oil, phytosterol removal, Trisyl^®^, vacuum distillation, polymers, tocopherols, *trans* fatty acids

## Abstract

Phytosterols in vegetable oils have gained attention for their nutritional benefits in foods and food supplements. However, the use of vegetable oils in emulsions for infant formulas and parenteral nutrition has raised some concerns, as phytosterols may contribute to phytosterolemia in the case of infant formulas and, in a second scenario, to parenteral nutrition-associated liver disease. The present study proposes removing phytosterols from soybean oil using a synthetic amorphous silica Trisyl^®^ (E551) as an adsorbent material. The process is simple and involves stirring the oil at a high temperature under vacuum conditions followed by filtration to remove the adsorbent. A rotational factorial design of experiments, considering the adsorbent/oil ratio, temperature, and time was carried out to determine the optimal conditions. Additionally, the effects on tocopherols levels and formation of *trans* fatty acids were explored. The total sterol content in the initial refined soybean oil was 2540 mg/kg, with 32% in ester form (813 mg/kg). The treatments effectively reduced the sterol concentration, achieving a reduction of nearly 70% when 10% Trisyl^®^, 140 °C, and a 90-min treatment were applied. Under these conditions, nearly 80% of the oil was recovered. Campesterol and stigmasterol levels were almost halved. Tocopherol losses were found to be below 20%. Thermal degradation, as analyzed by triacylglycerol polymers and *trans* fatty acids, was not observed in the treatments.

## 1. Introduction

Edible fats and oils are mainly composed of triacylglycerols and a minor fraction of components of a different chemical nature, known as the unsaponifiable fraction, which includes sterols, hydrocarbons, tocopherols, and others. The sterols can be found in a “free” form or as conjugates in which the hydroxy group is esterified or glycosylated (usually with glucose and rarely with other sugars). The four common classes of conjugated sterols are esters with fatty acids (SE), esters with hydroxycinnamic acids (HSE), such as ferulic or *p*-coumaric acid, glucosides (SG), and acylated glucosides (ASG) [1].

Phytosterols (PS) in vegetable oils have gained attention for their cholesterol-lowering effects and other health benefits [2]. Chemically similar to cholesterol, PS differ mainly in their side chains, forming a diverse family of compounds. PS have been proposed as dietary supplements to lower plasma LDL cholesterol (LDL-C). However, concerns have been raised regarding their potential atherogenic effects, which could counterbalance their benefits [3]. Phytosterolemia [4,5], linked to elevated plasma PS concentrations after consuming PS-enriched foods, has been associated with severe atherosclerosis, particularly in children and adolescents. This raises concerns that phytosterols (PS) may exacerbate atherosclerosis even more than LDL-C [6]. These complications seem to decrease in frequency with age [7].

Infant nutrition plays a crucial role in determining blood cholesterol levels and the risk of cardiovascular disease (CVD) later in life [8]. Research suggests that breastfeeding may be linked to lower blood cholesterol levels in adulthood [9]. Human milk contains high levels of cholesterol, whereas most infant formulas, which rely on vegetable oils, have significantly lower cholesterol levels. These formulas not only offer reduced cholesterol but also include PS, which are known to inhibit cholesterol absorption [10]. The PS content in infant formulas has been reported to range from 3.1 to 5.0 mg/100 mL [11], a notable contrast to the much lower levels in human milk (0.1 mg/100 mL) [12]. As a result, formula-fed infants often increase endogenous cholesterol synthesis to compensate for the low dietary intake of cholesterol [13,14]. Some experts recommend that infant formulas for children under five should either eliminate PS or reduce their concentration while increasing cholesterol content [13,15]. Currently, no formal guidelines exist regarding the maximum allowable PS concentration in infant formulas.

Similar concerns have arisen regarding the use of vegetable oils in the lipid phase of parenteral lipid-based emulsions (PLE). High concentrations of PS in the lipid fraction of PLE are suspected to contribute to parenteral nutrition-associated liver disease (PNALD), particularly in infants [16,17]. Nandivada et al. [18] reviewed evidence on the effects of PS levels in intravenous lipid emulsions on PNALD, showing that strategies to reduce soybean oil, which has high PS content, and use oil blends instead, lower PS concentrations from 550–600 mg/kg to 225–250 mg/kg. Despite these findings, no official guidelines, including those from the FDA, currently regulate PS levels in PLE.

Sterols can be removed from fat matrices by applying different technological approaches. Numerous references document the extraction of cholesterol from animal fats [19,20,21]. Notably, Wang [22] conducted a significant study on the removal of cholesterol from marine oils using the Trisyl^®^ additive [23]. The food additive Trisyl^®^ is an amorphous synthetic form of silicon dioxide produced under controlled conditions for specific adsorptive properties. It is an odorless, tasteless, free-flowing white powder with over 99.0% silicon dioxide purity when dry. Trisyl^®^ is known to be a selective and efficient adsorbent of phosphatides, trace metals (especially copper and iron), soaps, and lipid oxidation compounds. Compared to some activated bleaching earth with a pH of 2 to 3, Trisyl^®^ has a less acidic pH of 4.5 [24]. Wang [22] proposed a solid–liquid extraction treatment with Trisyl^®^, bleaching earth, or a mixture of the two. The process involves heating the oil in the presence of the adsorbent at temperatures ranging from 100 °C to 210 °C (preferably 150 °C to 170 °C) for more than one minute, optionally at a pressure below 133 Pa (1 mm Hg), ideally less than 1.33 Pa [22].

The processing of oils can also modify the structure of triacylglycerols by polymerization at high temperatures and affect the content of other compounds of nutritional impact such as tocopherols and *trans* fatty acids (TFA) [25].

Soybean oil contains the highest amounts of tocopherols among common plant oils. The contents of α-, β-, γ-, and δ-tocopherols decrease during refining, with significant losses occurring especially in the deodorizing stage [26]. Tocopherols are essential components of parenteral formulas due to their antioxidant properties and their ability to protect other nutrients and cells in the body, thereby contributing to improved patient health and recovery. It has been demonstrated that the incorporation of α-tocopherol into fish oil enhances the anti-inflammatory response to endotoxins, compared to fish oil-based intravenous lipid emulsions alone, while also preventing parenteral nutrition-induced liver injury and essential fatty acid deficiency in mice [27].

*Trans* fatty acids (TFA) can be formed through the isomerization of cis unsaturated fatty acids (UFAs) from either natural sources, such as enzymatic hydrogenation or biohydrogenation, or industrial processes, particularly partial catalytic hydrogenation. A growing body of evidence indicates that the majority of TFA are associated with adverse health outcomes, including an elevated risk of cardiovascular diseases, inflammatory processes, cancer, and other medical conditions [28]. TFA formation during the deodorization step is well established [29]. Additionally, inadequate neutralization or bleaching can lead to increased TFA levels [30].

This study aims to reduce the levels of phytosterols in soybean oil for use in infant formula and parenteral nutrition by exploring the potential of Trisyl^®^. A rotational experimental design was employed, with independent variables including the adsorbent/oil ratio, temperature, and time. Additionally, the effects on tocopherol levels, isomerization of fatty acids and polymers were also examined to determine the extent of chemical degradation during the treatments.

## 2. Materials and Methods

### 2.1. Samples, Standars and Reactants

Refined soybean oil (Fresenius Kabi AB, Brunna, Sweden) and Trisyl^®^ Sylica (Grace, Columbia, MD, USA) were used as received. Trisyl^®^‘s main characteristics are shown as Supplementary Materials (Table S1).

5-α-cholestan-3β-ol and cholesteryl stearate of chromatographic purity higher than 99% (Sigma-Aldrich, St. Louis, MO, USA) were used. These standards were used as internal standards and to construct calibration lines to evaluate the response factors of the groups of compounds of interest. The solutions were prepared in a concentration range of 5–120 μg/mL.

All reagents and solvents used were of analytical grade. Diethyl ether, ethanol, chloroform, pyridine, and potassium hydroxide were provided by Panreac (Barcelona, Spain). 

Trimethylchlorosilane (TMCS) and hexamethyldisiloxane (HMDS) were obtained from Supelco (Park Bellefonte, PA, USA). The silanization reagent was pyridine:HMDS:TMCS, 9:3:1.

### 2.2. Design of Experiments

Response Surface Methodology (RSM) has been used as a regression model to analyze the significance of the experimental data. A rotating composite design has been used to consider the combined effect of the dependent variables to determine the optimal conditions, providing a wider and more reliable range for observing trends. The 2^3^ factorial design was used, consisting of 8 assays (−1 and +1), 3 center points, and 6 axial points (−1.68 and +1.68), and it resulted in an orthogonal distribution for a total of 17 experiments [31].

Following Wang’s study [22] on cholesterol reduction in marine oils, three variables were selected: time, temperature, and additive amount (expressed as the weight percentage of the additive relative to the oil). The range for each variable was chosen based on Wang’s [22] optimal values. Table 1 shows the relationship between coded variables and the values used in each trial. The experimental data were then collected and analyzed statistically by fitting second-order polynomial models. 

### 2.3. Treatments

The treatments were conducted under a vacuum (<2 mbar) using a discontinuous distillation device with nitrogen stripping. For each experiment, a quantity of 200 g of soybean oil was placed in a round-bottomed, three-necked flask, which was heated with a hemispherical heating mantle. The heating mantle was connected to a PID controller with a thermoregulator and a thermocouple. The flask was connected to a nitrogen source, regulated by a rotameter and a vacuum pump, with cold traps in between. The treatments were performed under a vacuum pressure of lower than 2 mbar. When the target temperature was reached, the adsorbent Trisyl^®^ was added, marking the start of the treatment, and the time was recorded. Nitrogen was injected when the oil sample reached 100 °C to ensure effective heat transfer and agitation, with a gas flow set to 45 mL/min. After the treatment, the oil was cooled to 40 °C, and the vacuum was released with nitrogen to protect the product. The oil was then filtered through filter paper (pore size, 35 μm, Esteryfil 123, Barcelona, Spain) to remove the adsorbent and stored at 4 °C until analysis. 

To establish the mass balance, the oil retained in the vessels was extracted using hexane and combined with the oil from the paper filter and solid residue, which was extracted in a Soxhlet for six hours. The solvent was then evaporated under a vacuum until dry, and the remaining oil retained in residues (ORR) was weighed. The sterol content was also analyzed in the extracted oil.

### 2.4. Sterol Analysis

The contents of the individual and total sterols were determined in the filtered oil and in the oil extracted from the solid residues, according to ISO 12228-1 [32]. Basically, the oil sample with 5-α-cholestan-3β-ol added as an internal standard was saponified with potassium hydroxide in an ethanolic solution, and the unsaponifiable fraction was then extracted with ethyl ether. The sterol fraction was separated from the unsaponifiable extract by thin layer chromatography using a basic silica gel plate. The sterols recovered from the silica gel were transformed into trimethylsilyl ethers and analysed by gas chromatography with flame ionisation detection (GC-FID).

For the analysis of sterol esters, the oil was subjected to column adsorption chromatography using hydrated silica gel, with cholesteryl stearate used as internal standard [33]. The fraction eluted under the test conditions was recovered, and basic conditions (transmethylation with potassium methylate 2M) were applied to transform the sterol esters into their free forms. Without further fractionation, the extract was dried, silylated to transform the free sterols into trimethyl-silyl ethers, and analysed by GC-FID in a manner entirely analogous to the analysis of total sterols.

### 2.5. Other Determinations

The contents of tocopherols, trans-unsaturated fatty acids and polymers were determined in initial and filtered oils by IUPAC methods [34,35,36].

### 2.6. Statistical Analysis of Data

To optimize and understand the influence of the variables under investigation, this study performed a statistical analysis of data from all 17 experiments by an Analysis of Variance (ANOVA) combined with Response Surface Methodology (RSM) to evaluate the model response using a second-order polynomial and to determine the significance of the variables. All statistical analyses were carried out with Statistica v.7 (Stat-Soft Inc., Tulsa, OK, USA).

## 3. Results and Discussion

### 3.1. Mass Balance and Sterol Content in Oils and Solid Residues

The initial refined soybean oil had a total sterol content of 2540 mg/kg, with 32% (813 mg/kg) in esterified form. Table 2 shows the mass balance and the analysis of sterols in the oil treated (filtered oil) and the oil extracted from the adsorbent (solid residue). On average, 83.5% (±3.5%) of the initial oil was recovered as filtered oil. The amount of oil retained in the solid residue was directly correlated with the amount of additive used, i.e., the greater the amount of additive, the higher the oil retention.

From an economic perspective, this unconventional process results in significant oil losses (between 11.4–22.6%, depending on the adsorption conditions), and in case the adsorbent cannot be reused, the cost of the obtained oil would be increased. However, given that this process has been designed to meet the potential needs of the pharmaceutical industries, the added value of the final product may offset the above-mentioned increase in the cost of production.

The total sterol content decreased in all filtered oil samples (Table 2), with sterol levels being 40–69% lower than in the original oil. The best results were obtained in experiment 2, where conditions of 140 °C, 10% adsorbent, and 90 min were applied. Under these conditions, almost 80% of the oil was recovered. The reduction in esterified esters was not proportional to the decrease in total sterols, mainly due to their poor adsorption onto the adsorbent (Table 2). Additionally, sterol esterification was even observed during the treatments, leading to a substantial change in the ratio between the free and esterified sterols in the oil.

As expected, the oil samples extracted from the solid residue displayed higher sterol contents than the original oil due to a concentration effect. On average, 50% of the expected amount of sterols in the extracted oil was undetected, with losses ranging from 29.1% in experiment 1 to 63.8% in experiment 14. As discussed below, this discrepancy may be due to partial losses by distillation as a consequence of the relatively high temperature and vacuum conditions applied [37] and/or chemical transformations (esterenes) by acid-catalysed dehydration on the adsorbent [38]. In addition, the unexpected levels of sterols in the oil extracted from the solid residue could also be attributed to an inefficient capability of hexane to extract strongly adsorbed sterols, requiring a more polar solvent for complete extraction [39].

### 3.2. Effects of the Different Operation Conditions on the Total Content of Sterols

**Figure 1:**
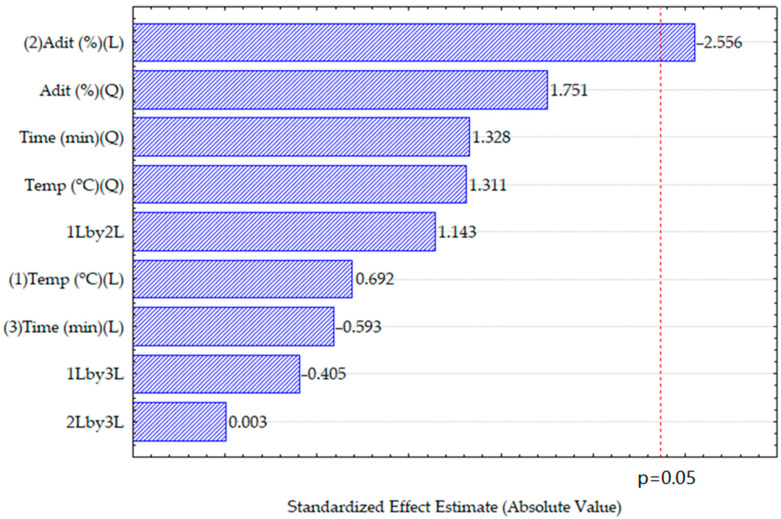
Pareto chart for the standardized effects of the variables and interactions on total sterols in filtered oils. (1), (2), and (3) represent the factors of temperature, amount of adsorbent, and time, respectively, while (L) and (Q) stands for linear and quadratic models, respectively. 1Lby2L, 1Lby3L, and 2Lby3L represent the interactions between the factors in a linear model. For instance, 1Lby2L denotes the interaction between temperature and the amount of adsorbent.

The results of the ANOVA indicated that 65.13% of the total variability in the data was explained by the factors included in the model (Table S2). The proportion of the additive Trisyl^®^ had the most significant impact on reducing the content of sterols, while time and temperature had a limited effect within the range of variables studied, as shown in the Pareto diagram (Figure 1).

**Figure 2:**
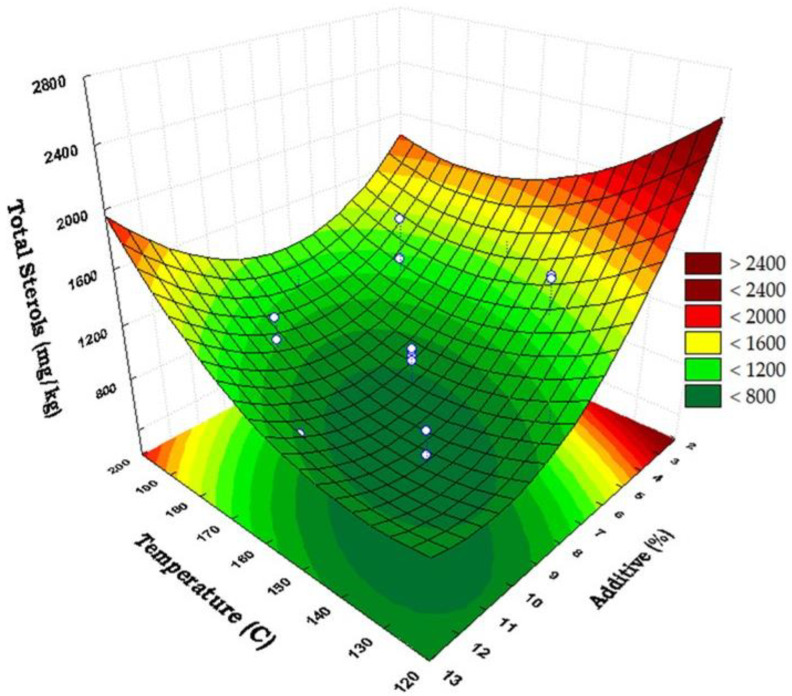
Response surface of total sterols’ content (mg/kg) in the treated oil versus temperature (°C) (y) and the additive Trisyl^®^ (%) (x) at 60 min. White bullets correspond to experimental data.

Wang [22] reported that at 80 °C, 3% clay reduced cholesterol by approximately 15%, whereas at 180 °C, the same concentration of clay removed over 60% of the cholesterol from the oil. Furthermore, using 6% sorbent at 190 °C for 10–20 min, cholesterol levels in fish oil were reduced to between 0.5 and 0.8 mg/g, with most treatments being conducted for 20 min. From our results, the interaction between the amount of additive and temperature was also not significant on the final content of sterols. A data analysis was conducted on the response surface of these two factors to determine the conditions that resulted in the highest sterol losses in the oil (Figure 2). 

The analysis showed that the minimum value of total sterols in the oil would be 698 mg/kg, i.e., a reduction of 72.5% of the initial content, when applying 145.9 °C, 9.8% of the additive, and 62.2 min of treatment (Figure 2). The findings of this study are consistent with previously reported data [37,38,40,41,42,43], highlighting similarities between the oil refining process and the treatment under investigation. Specifically, the treatments resembled the bleaching stage of oil refining, where acid-activated solids are employed to remove the color compounds, and the deodorization step, which involves a vacuum, nitrogen stripping, and high temperatures. In this context, several authors have reported a slight increase in the free sterol content during the bleaching process, attributing it to the acid-catalyzed hydrolysis of steryl esters [37,38,39,40,41,42,43]. During bleaching, a slight reduction in total sterol content has also been observed, attributed to the formation of sterenes, with the extent of this transformation depending on operating conditions [37,38,41]. For instance, increasing the bleaching temperature from 90 to 110 °C resulted in a gradual rise in sterene concentration from 5.1 to 22.3 mg/kg. The formation of sterenes was significantly increased with prolonged bleaching time, higher concentrations of bleaching earth, and enhanced acid activation. Additionally, the formation of these nonpolar steroids during bleaching was found to be directly proportional to the content of esterified sterols, which dehydrated more readily than free sterols [40]. Bai et al. [38] demonstrated that the loss of free sterols from corn oil was not only due to their adsorption on activated clay, but also due to the degradation of those sterols adsorbed at sites with acidic characteristics. During the deodorization and physical refining processes, free sterols are also dehydrated into sterenes, with reported values ranging from 0.46% to 1.06% depending on temperature [41]. Along with free sterols, the sterenes formed are subsequently distilled and recovered in the deodorizer distillate.

As to the sterol composition, the levels of campesterol and stigmasterol were significantly reduced, while Δ7-sterols tended to increase with the treatments due to sterol isomerization (Table 3). These findings align with previous reports [42,43]. Structural changes in sterols can be attributed to acidic conditions and rigorous bleaching processes [44,45]. Recently, Bai et al. [38] confirmed free sterols degradation in bleaching using a first-order kinetic model, and the degradation susceptibility was as follows: stigmasterol > campesterol > β-sitosterol. The presence of oxygen and activated clay promotes the degradation of free sterols and the formation of their derivatives, including sterenes.

The campesterol and stigmasterol levels were also lower in the oil from solid residues than those in the initial oil (Table 4), but these differences were not substantial compared to those found in the filtered oil. 

### 3.3. Effects of the Different Operation Conditions on Tocopherols, Polymerization, and Fatty Acid Isomerization

The effects on other compounds of nutritional interest, such as tocopherols, were studied in selected trials. On average, a reduction of 18% in tocopherol content was observed when a 10% additive was used with a treatment time of 10 min (Table 5). Although some studies have reported a significant impact of temperature on tocopherol depletion in the bleaching step of oil refining, particularly within the temperature range of 90 °C to 145 °C [46], our trials showed that the total loss of tocopherols was primarily influenced by the additive content and the duration of treatment. The high vacuum maintained during these trials may have further contributed to tocopherol loss through distillation.

Among the different tocopherols, δ-tocopherol exhibited the greatest decline in all cases, with a maximum loss of 37.1% observed at the highest temperature. This finding is consistent with previous studies showing that δ-tocopherol and γ-tocopherol are less stable under oxidative conditions [47].

Regarding polymers and *trans* fatty acids, none of the variables studied significantly affected the levels of these groups of compounds within the ranges considered (Table 6). The overall fatty acid composition remained unaffected by the treatments (Table S3). 

Previous studies have investigated TFA formation during deodorization with nitrogen as the stripping gas [48]. The amount of TFA generated during the deodorization or physical refining of edible fats was influenced primarily by temperature and process duration, particularly at higher temperatures and longer durations than those used in these assays. The total TFA content increased as these factors were intensified. This is particular important due to the regulatory limit on *trans* fatty acid content in oils intended for use in PLE formulations. Products labelled as “free from *trans* fatty acids” must not exceed 1 g of *trans* fatty acids per 100 g of oil or fat. In this regard, the desterolized oils obtained in the present study complied with the limit imposed in PLE formulations, with *trans* fatty acid levels below 0.85% in all cases.

## 4. Conclusions

From the experimental results, it can be concluded that the total initial sterol content of soybean oil can be drastically reduced (by 40–69% compared to the original oil) during the bleaching-like process within the parameter window selected in this study. The greatest depletion was achieved under the following conditions: a bleaching temperature of 140 °C, 10% adsorbent/oil ratio, and a treatment time of 90 min. Under these conditions, almost 80% of the oil was recovered. In addition, the reduction in esterified esters was not proportional to the decrease in total sterols, leading to a substantial change in the ratio between free and esterified sterols in the oil. Under the optimal processing conditions, the tocopherol level in the initial soybean oil was reduced by 20%. Within the ranges of the variables studied, no significant thermal degradation as evaluated by polymers or *trans* fatty acids was observed. 

## Figures and Tables

**Table 1:** Design matrix for three factors central composite design and the relationship between the coded variables and the values taken in each run.

RUN	X1	X2	X3	Temperature (°C)	Additive (% *w*/Oil *w*)	Time (min)
1	−1.0	−1.0	−1.0	140	5.00	30.0
2	−1.0	1.0	1.0	140	10.00	90.0
3	1.0	−1.0	1.0	180	5.00	90.0
4	1.0	1.0	−1.0	180	10.00	30.0
5 (C)	0.0	0.0	0.0	160	7.50	60.0
6	−1.0	−1.0	1.0	140	5.00	90.0
7	−1.0	1.0	−1.0	140	10.00	30.0
8	1.0	−1.0	−1.0	180	5.00	30.0
9	1.0	1.0	1.0	180	10.00	90.0
10 (C)	0.0	0.0	0.0	160	7.50	60.0
11	−1.7	0.0	0.0	127	7.50	60.0
12	1.7	0.0	0.0	193	7.50	60.0
13	0.0	−1.7	0.0	160	3.32	60.0
14	0.0	1.7	0.0	160	11.68	60.0
15	0.0	0.0	−1.7	160	7.50	9.8
16	0.0	0.0	1.7	160	7.50	110.2
17 (C)	0.0	0.0	0.0	160	7.50	60.0

(C) represents the central point of the experimental design.

**Table 2:** Total sterol content (mg/kg) and esterified sterols (mg/kg) in filtered oils (FO) and the ones obtained from the solid residues (ORR).

RUN	Filtered Oil (FO)			Oil Retained in Residues (ORR)		
	Yield * (% *w/w*)	Total Sterols (mg/kg)	Esterified Sterols (mg/kg)	Yield * (% *w/w*)	Total Sterols (mg/kg)	Esterified Sterols (mg/kg)
1	85.4	1511	839	13.1	3730	978
2	78.0	786	613	19.7	1533	689
3	87.2	1207	790	12.2	2689	807
4	80.1	1209	834	19.4	2550	780
5 (C)	80.6	939	568	15.2	2211	618
6	85.9	1493	517	12.2	4019	575
7	81.4	960	790	15.7	2386	552
8	88.6	1522	1004	10.3	3732	800
9	81.3	1052	792	16.6	1774	699
10 (C)	82.8	924	931	15.5	2476	596
11	83.5	805	647	6.2	3724	593
12	84.4	1034	673	7.0	1977	659
13	89.1	1183	720	8.7	3840	814
14	77.4	837	533	22.7	1489	751
15	82.5	885	566	15.2	2503	283
16	85.6	962	510	13.8	2417	486
17 (C)	86.2	979	906	12.5	2367	734

* Percentage of oil relative to the initial amount. The total sterol content of the initial refined soybean oil was 2540 mg/kg (with 813 mg/kg in ester form). (C) denotes the central point of the experimental design.

**Table 3:** Composition of Total Sterols (%) in Initial and Filtered Oil samples.

Sterols (%)	Campest.	Stigmast.	Δ7- Campst.	β- Sitost.	Δ5- Avenast.	Δ5,24- Stigmast.	Δ7- Stigmast.	Δ7- Avenast.	Others
INITIAL	20.02	16.20	0.42	54.14	2.08	1.33	2.91	1.72	1.14
1	14.63	13.43	0.75	58.67	1.94	1.38	5.23	3.07	0.92
2	9.15	7.24	n.d.	67.35	2.88	0.63	7.72	3.86	1.15
3	13.16	12.67	n.d.	63.06	2.38	n.d.	4.70	2.47	1.57
4	11.53	10.68	n.d.	59.61	3.76	2.05	7.32	3.20	1.84
5 (C)	9.57	6.93	n.d.	77.57	0.80	0.68	3.56	0.87	0.00
6	13.82	11.23	0.45	63.91	2.70	2.66	1.36	1.20	2.67
7	12.23	10.48	0.21	64.59	2.71	4.22	3.72	1.02	0.82
8	13.40	12.38	1.17	60.58	3.82	4.65	2.04	0.93	1.03
9	10.26	7.58	0.80	65.05	3.94	6.26	2.90	2.90	0.31
10 (C)	13.64	9.40	1.01	59.60	3.90	4.92	2.58	2.29	2.68
11	12.39	11.08	n.d.	65.32	2.21	0.55	4.94	2.41	1.10
12	11.47	9.60	n.d.	66.34	1.75	0.86	5.77	3.01	1.20
13	14.74	14.63	n.d.	61.57	1.96	0.33	3.66	1.83	1.27
14	9.46	6.97	n.d.	68.30	1.94	1.35	7.05	3.56	1.37
15	12.57	11.38	n.d.	63.53	2.25	0.78	5.10	2.79	1.60
16	10.66	9.22	0.48	66.48	2.64	3.14	5.04	2.17	0.17
17 (C)	9.85	8.12	n.d.	66.78	1.78	6.50	3.33	2.57	1.06

n.d.—not detected.

**Table 4:** Composition of total sterols (%) in the initial oil and in the retained oil in the residues.

Sterols (%)	Campest.	Stigmast.	Δ7-Campst.	β- Sitost.	Δ5- Avenast.	Δ5,24- Stigmast.	Δ7- Stigmast.	Δ7- Avenast.	Others
INITIAL	20.02	16.20	0.42	54.14	2.08	1.33	2.91	1.72	1.14
1	19.59	19.75	n.d.	55.53	0.14	1.07	2.31	1.21	0.40
2	15.11	14.43	n.d.	61.46	3.00	0.50	3.29	1.46	0.76
3	17.79	17.47	n.d.	56.86	2.34	0.29	2.32	1.06	1.86
4	18.20	17.70	n.d.	57.65	1.93	0.30	2.17	0.88	1.17
5 (C)	15.20	14.78	n.d.	61.67	3.05	1.29	2.57	1.03	0.41
6	19.35	18.25	n.d	56.67	3.66	0.21	1.18	0.50	0.20
7	16.91	18.24	n.d	57.69	2.58	1.35	1.37	0.44	1.20
8	16.04	17.85	n.d	57.96	2.54	1.48	1.96	0.46	2.64
9	15.17	14.58	n.d	60.77	2.83	1.60	2.70	0.31	1.35
10 (C)	17.67	16.55	n.d	56.58	2.51	2.19	2.21	0.91	1.15
11	18.84	18.76	n.d.	57.43	1.59	0.44	1.35	0.72	0.44
12	16.01	15.23	n.d.	60.86	1.84	0.63	2.88	1.41	0.68
13	18.54	18.64	n.d.	57.58	1.59	0.33	1.66	0.78	0.55
14	14.01	13.78	n.d.	62.41	2.10	0.63	3.77	1.83	0.90
15	19.86	19.07	n.d.	57.22	0.45	1.62	0.94	0.41	0.43
16	18.25	16.60	0.58	57.68	2.10	0.64	2.10	1.43	0.34
17 (C)	17.33	17.19	n.d.	60.00	1.47	0.32	2.34	1.21	0.14

**Table 5:** Tocopherols (mg/kg) of the filtered oil samples obtained after the treatments.

RUN	α-toc	β-toc	γ-toc	δ-toc	TOTAL	Loss (%)
INITIAL	94	24	389	124	630	
1	89	22	374	116	602	4.4
2	79	20	313	92	504	20.0
3	89	21	355	103	567	10.0
4	85	23	329	85	521	17.3
5 (C)	86	22	334	99	541	14.1
11	91	24	371	114	600	4.8
12	86	18	314	78	496	21.3
13	94	24	374	116	608	3.5
14	80	18	295	86	479	24.0
15	91	24	359	110	585	7.1
16	88	21	329	98	536	14.9
17 (C)	89	21	336	100	546	13.3

**Table 6:** Percentage of the polymers and *trans* fatty acids in the filtered oil samples obtained after various treatments.

RUN	Polymers (%)	*∑trans* FA (%)
INITIAL	0.4	0.75
1	0.3	0.74
2	0.6	0.76
3	0.6	0.76
4	0.4	0.84
5 (C)	0.6	0.80
11	0.5	0.84
12	0.5	0.82
13	0.4	0.83
14	0.3	0.80
15	0.3	0.76
16	0.4	0.78
17 (C)	0.6	0.77

## Data Availability

The original contributions presented in the study are included in the article/supplementary material, further inquiries can be directed to the corresponding author.

## Supplementary Materials

The following supporting information can be downloaded at: https://www.mdpi.com/article/10.3390/foods13193172/s1, Table S1: Trisyl^®^ characteristics; Table S2: Statistical parameters obtained from the analysis of variance for Total Sterols (mg/kg) as dependent variable; Table S3: Fatty acid composition (%) of the oil samples obtained after the treatments.
