# Supplementary material for: Phytosterol Depletion in Soybean Oil Using a Synthetic Silica Adsorbent
Source: Foods. 2024 Oct 6;13(19):3172. doi: 10.3390/foods13193172 (PMC11475823; doi:10.3390/foods13193172)
Supplement: Supplementary file 1 [file foods-13-03172-s001.zip › foods-3225218-supplementary.pdf]

Table S1.-Trisyl® characteristics

Moisture content (%)	58.19 ± 1.44
Sodium (%)	0.014 ± 0.001
Sulfur (%)	0.009±0.0
Potassium (%)	0.007 ± 0.001
Calcium (%)	0.011 ± 0.0
Phosphorus (%\	0.001 ±:0.0
Magnesium (%)	0.002 ± 0.001
Surface area (m^2/g)	462.75±0.274
Pore diameter (Å)	50.69 ± 0.057
Pore volume (cm_3/g)	0.625 ±0.003

Table S2.- Table 4. Statistical parameters obtained from the analysis of variance for Total Sterols (mg/kg) as Dependent Variable

$R^2 = 0.6513$; R^2 Adj, 0.20298 ; MS Residual, 59803.85

Factors	SS	df	MS	F	p
(1)Temperature (°C)(L)	28648	1	28648.1	0.479034	0.511159
Temperature (°C)(Q)	102793	1	102792.7	1.718831	0.231212
(2)Additive (%)(L)	390708	1	390708.4	6.533164	0.037771
Additive (%)(Q)	183299	1	183298.6	3.064997	0.123464
(3)Time (min)(L)	21058	1	21058.1	0.352120	0.571583
Time (min)(Q)	105484	1	105483.9	1.763831	0.225813
1L by 2L	78210	1	78210.1	1.307777	0.290394
1L by 3L	9800	1	9800.0	0.163869	0.697704
2L by 3L	0	1	0.5	0.000008	0.997774
Error	418627	7	59803.8		
Total SS	1200544	16			

Continuation Table S2 -Effect Estimates

Factor	Effect	Std.Err.	t(7)	p	-95,%	+95,%	Coeff.	Std.Err.
Mean/Interc.	794.604	140.8120	5.64302	0.000780	461.637	1127.572	794.604	140.8120
(1)Temperature (°C)(L)	91.793	132.6249	0.69212	0.511159	-221.815	405.401	45.896	66.3125
Temperature (°C)(Q)	192.224	146.6193	1.31104	0.231212	-154.475	538.923	96.112	73.3096
(2)Additive (%)(L)	-338.990	132.6249	-2.55601	0.037771	-652.598	-25.382	-169.495	66.3125
Additive (%)(Q)	256.688	146.6193	1.75071	0.123464	-90.011	603.388	128.344	73.3096
(3)Time (min)(L)	-78.699	132.6249	-0.59340	0.571583	-392.307	234.909	-39.350	66.3125
Time (min)(Q)	194.724	146.6193	1.32809	0.225813	-151.975	541.423	97.362	73.3096
1L by 2L	197.750	172.9217	1.14358	0.290394	-211.145	606.645	98.875	86.4609
1L by 3L	-70.000	172.9217	-0.40481	0.697704	-478.895	338.895	-35.000	86.4609
2L by 3L	0.500	172.9217	0.00289	0.997774	-408.395	409.395	0.250	86.4609

Abbreviations: R^2, Correlation coefficient, dF, degrees of freedom; SS, sum of squares due to the source; MS, mean sum of squares due to the source; F, F-statistic; p, p-value; t, *t* Students; L/Q stands for linear and quadratic models.

Table S3 - Fatty Acid Composition (%) of the oil samples obtained after the treatments.

Fatty Acid (%)	C16:0	C18:0	C18:1	C18:2	C18:3	C20:0	C20:1	C22:0	C24:0	∑*trans*
INITIAL	10.44	2.99	25.03	53.77	5.86	0.32	0.24	0.43	0.16	0.75
1	10.44	3.01	24.80	53.97	5.88	0.32	0.24	0.44	0.16	0.74
2	10.37	3.00	24.86	53.97	5.88	0.32	0.24	0.44	0.16	0.76
3	10.41	3.01	24.82	53.97	5.87	0.32	0.24	0.44	0.16	0.76
4	10.48	2.99	24.78	53.91	5.89	0.31	0.23	0.41	0.15	0.84
5 (C)	10.42	3.01	24.85	53.90	5.87	0.32	0.24	0.43	0.16	0.80
11	10.39	3.01	24.88	53.86	5.86	0.32	0.24	0.44	0.17	0.84
12	10.46	3.01	24.86	53.87	5.85	0.32	0.24	0.42	0.15	0.82
13	10.47	3.03	24.84	53.80	5.86	0.32	0.24	0.44	0.17	0.83
14	10.40	3.01	24.88	53.87	5.87	0.33	0.24	0.43	0.16	0.80
15	10.43	3.02	24.58	54.12	5.90	0.32	0.24	0.44	0.17	0.76
16	10.41	3.02	24.86	53.88	5.87	0.33	0.24	0.44	0.17	0.78
17 (C)	10.39	3.01	24.85	53.93	5.88	0.33	0.24	0.44	0.16	0.77
